# Differential regulation of NF-κB activation and function by topoisomerase II inhibitors

**DOI:** 10.1186/1471-2407-6-101

**Published:** 2006-04-21

**Authors:** Kirsteen J Campbell, John M O'Shea, Neil D Perkins

**Affiliations:** 1 School of Life Sciences, Division of Gene Regulation and Expression, MSI/WTB, Complex, Dow Street, University of Dundee, Dundee, DD1 5EH, Scotland, UK

## Abstract

**Background:**

While many common chemotherapeutic drugs and other inducers of DNA-damage result in both NF-κB nuclear translocation and DNA-binding, we have previously observed that, depending on the precise stimulus, there is great diversity of the function of NF-κB. In particular, we found that treatment of U-2 OS osteosarcoma cells with the anthracycine daunorubicin or with ultraviolet (UV-C) light resulted in a form of NF-κB that repressed rather than induced NF-κB reporter plasmids and the expression of specific anti-apoptotic genes. Anthracyclines such as daunorubicin can induce DNA-damage though inhibiting topoisomerase II, intercalating with DNA and undergoing redox cycling to produce oxygen free radicals. In this study we have investigated other anthracyclines, doxorubicin and aclarubicin, as well as the anthracenedione mitoxantrone together with the topoisomerase II inhibitor ICRF-193, which all possess differing characteristics, to determine which of these features is specifically required to induce both NF-κB DNA-binding and transcriptional repression in U-2 OS cells.

**Results:**

The use of mitoxantrone, which does not undergo redox cycling, and the reducing agent epigallocatechingallate (EGCG) demonstrated that oxygen free radical production is not required for induction of NF-κB DNA-binding and transcriptional repression by these agents and UV-C. In addition, the use of aclarubicin, which does not directly inhibit topoisomerase II and ICRF-193, which inhibits topoisomerase II but does not intercalate into DNA, demonstrated that topoisomerase II inhibition is not sufficient to induce the repressor form of NF-κB.

**Conclusion:**

Induction of NF-κB DNA-binding and transcriptional repression by topoisomerase II inhibitors was found to correlate with an ability to intercalate into DNA. Although data from our and other laboratories indicates that topoisomerase II inhibition and oxygen free radicals do regulate NF-κB, they are not required for the particular ability of NF-κB to repress rather than activate transcription. Together with our previous data, these results demonstrate that the nature of the NF-κB response is context dependent. In a clinical setting such effects could profoundly influence the response to chemotherapy and suggest that new methods of analyzing NF-κB function could have both diagnostic and prognostic value.

## Background

In mammalian cells, the NF-κB family of transcription factors is composed of homodimers and heterodimers derived from five distinct subunits, RelA(p65), c-Rel, RelB, p50 (NF-κB1) and p52 (NF-κB2) [1]. Of these, p50 and p52 arise from proteolytic degradation of larger precursor proteins, p105 and p100 respectively. In unstimulated cells, the majority of NF-κB complexes are kept predominantly cytoplasmic and in an inactive form by binding to a family of inhibitory proteins, the IκBs. Activation of NF-κB typically involves the phosphorylation of IκBs by IκB kinase (IKK) β (IKK2), a component of the IKK complex, which includes one other catalytic subunit, IKK α (IKK1), and a regulatory subunit IKK γ (NEMO) [1]. Many stimuli induce IKK activity through a variety of mechanisms [1]. Phosphorylation of IκB results in its ubiquitination and degradation by the proteasome. This frees NF-κB complexes to translocate to the nucleus. Aberrantly active NF-κB is associated with many human diseases, particularly those of an inflammatory origin [2]. Over the last few years, however, it has also become apparent that NF-κB plays critical roles in tumorigenesis and the response to cancer therapy [3,4].

Nuclear translocation and subsequent DNA-binding represent critical steps in the NF-κB pathway. However, the functional consequences of NF-κB activation, in terms of gene transcription, can differ dramatically depending on the nature of the inducer and the cellular context [4-6]. These differences derive from a wide variety of regulatory mechanisms that control the promoter targeting and transactivation functions of the NF-κB subunits [5]. Previously, we have demonstrated that the response of NF-κB to cytotoxic agents can exhibit great diversity [7,8]. While inflammatory stimuli such as tumor necrosis factor α (TNF) result in RelA-dependent induction of anti-apoptotic genes such as Bcl-xL and XIAP, other stimuli, such as treatment with ultraviolet light (UV-C) and the chemotherapeutic drug daunorubicin (also known as daunomycin) resulted in RelA-dependent transcriptional repression of these same genes [7]. These differences do not simply derive from the effects of DNA-damage. We also observed that the chemotherapeutic drug etoposide induced an activator form of NF-κB that behaved more similarly to TNF induced NF-κB [8]. Furthermore, treatment with the cancer drug cisplatin, which induces DNA-damage through DNA cross-linking, revealed that in the same U-2 OS osteosarcoma cell line used for the analysis of the other compounds, no induction of NF-κB DNA-binding occurred. Cisplatin, however, modulated RelA transcriptional activity, resulting in repression of Bcl-xL but not X-IAP expression [8]. Further analysis demonstrated that regulation of RelA transactivation by cisplatin shares many features with effects we had previously observed upon induction of the ARF tumor suppressor [8]. Together, these results reveal that the response of NF-κB to different cytotoxic agents and chemotherapeutic drugs, within the same tumor cell line, can demonstrate dramatic functional differences. Such differences could have consequences for the effectiveness of cancer treatment in patients and imply that improved diagnosis and choice of therapy might result from a more in depth knowledge of the mechanisms underlying these effects.

Although treatment with these chemotherapeutic drugs results in DNA-damage, this can occur through different mechanisms [9-11]. In addition, they often have other properties with the potential to result in specific biological effects (Table 1). For example, the anthracycline daunorubicin, which is primarily used in the treatment of acute myeloid leukemia and its congener doxorubicin (also known as adriamycin), which is used to treat a variety of solid tumours, osteosarcomas, soft-tissue sarcomas and non-Hodgkins lymphoma [12,13], can induce DNA-damage through inhibition of topoisomerase II [12]. These drugs inhibit the action of topoisomerase II after induction of double strand DNA breaks resulting in covalent attachment between active site residues of topoisomerase II and the 5' of the DNA ends [10,12,14]. Both daunorubicin and doxorubicin also intercalate into DNA and alter helical torsion, which also results in DNA-damage [10,12,14]. Thus, these compounds can essentially induce DNA-damage through 2 distinct mechanisms.

Daunorubicin and doxorubicin also undergo redox cycling and generate oxygen free radicals, which can have a variety of effects, including damage to cell membranes but also provides a third mechanism of inducing DNA-damage [12]. Indeed, free radical generation by anthracyclines is thought to be responsible for the cardiotoxicity that limits their therapeutic use [15,16]. NADPH-flavin reductase, cytochrome p450 reductase and mitochondrial NADH reductase can all reduce anthracyclines to a semiquinone radical [17]. This semiquinone radical can donate its free electron to molecular oxygen to generate the superoxide radical (O^•^_2_) [17]. Like hydrogen peroxide (H_2_O_2_), O^•^_2 _can generate hydroxyl radicals (^•^OH) upon interaction with metal ions [17]. This results in lipid peroxidation of plasma membranes, leading to a loss of mitochondrial inner membrane potential and consequent cytochrome c release and apoptosis. Reactive oxygen species can also directly damage DNA through generation of strand breaks and oxidized nucleic bases such as guanine to 8-hydroxyguanine, giving rise to G-T transversions [17]. UV-C also induces free radical formation by homolytic fission of hydrogen peroxide, inducing single strand DNA breaks [17]. Oxygen free radicals have been shown to induce NF-κB DNA-binding under some circumstances and could potentially represent a key component of the ability of cytotoxic agents to differentially regulate NF-κB function [18-20].

A feature of anthracyclines and other topoisomerase II inhibitors, is that, at least in part, these different characteristics derive from distinct molecular features and can be functionally separated. For example, the anthracyclines daunorubicin, doxorubicin and aclarubicin all share planar ring structures that intercalate into DNA [9,10,12]. The anthracenedione mitoxantrone also possesses a planar structure and intercalates with DNA while inhibiting topoisomerase II but unlike the anthracyclines does not undergo redox cycling to generate semiquinone radicals that result in free radical generation [10,16,21]. Mitoxantrone is therefore useful clinically since it results in reduced levels of cardiotoxicity [16]. Structural differences in aclarubicin (aclacinomycin A), which is used in the treatment of acute myelocytic leukemia, mean that it can intercalate into DNA, resutling in DNA-damage and prevention of topoisomerase II DNA-binding, but it does not stabilize a Topoisomerase II-DNA complex like the other anthracyclines [9]. Therefore, any DNA-damage induced does not result from double stranded DNA-breaks induced by inactivation of topoisomerase II.

Etoposide, an epipodophyllotoxin, is used to treat small cell lung cancers, leukemias, lymphomas and germ-line malignancies [10]. As described above, etoposide inhibits topoisomerase II activity resulting in DNA strand breaks but has the opposite effect to daunorubicin/doxorubicin on NF-κB, strongly stimulating NF-κB transcriptional activity [7,8]. Etoposide does not intercalate into DNA nor does it generate free radicals [10,14]. These observations all suggested that multiple factors determine NF-κB function in response to cytotoxic agents such as chemotherapeutic drugs and that induction of DNA-binding is a separable regulatory event from modulation of transactivation. Moreover, they indicate that it is not topoisomerase II inhibition and DNA-strand breaks *per se *that result in forms of NF-κB that repress transcription. Therefore, to help determine which characteristics of these compounds are required for different functions of NF-κB, we have investigated the response of NF-κB, both in terms of DNA-binding and transcriptional activity, to multiple topoisomerase II inhibitors with defined characteristics. In addition we have investigated the ability of an additional topoisomerase II inhibitor, the bis-dioxopiperazine compound ICRF-193, which is a catalytic inhibitor of topoisomerase II that stabilizes an inactive, closed clamp conformation of the enzyme that deprives the cell of topisomerase II catalytic activity, resulting in apoptosis without directly generating DNA breaks [22,23]. Furthermore, we have also analyzed the contribution of oxygen free radical generation to regulation of NF-κB function. Our results suggest that DNA-damage is required to induce NF-κB activity but whether this is achieved through DNA-intercalation or topoisomerase II inhibition will determine the function of the induced NF-κB complexes.

## Results

### Induction of NF-κB DNA-binding

All experiments in this report were performed in U-2 OS cells, a human osteosarcoma cell line. This provides continuity both within this investigation and with our previous published data [7,8]. It is likely, however, that results will vary in other cell lines and therefore while this data reveals the variability possible within a single tumour cell type, extrapolation to other cells should be performed with caution. Indeed, where possible, the effects of these drugs in different systems should be determined empirically.

**Figure 1:**
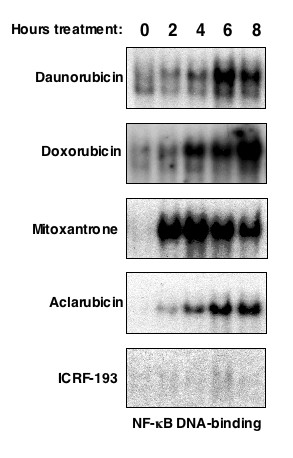
Induction NF-κB DNA binding by topoisomerase II inhibitors and DNA-intercalators. U-2 OS cells were stimulated with 1 μM aclarubicin, 1 μM daunorubicin, 1 μM doxorubicin, 2.5 μM mitoxantrone and 4 μM ICRF-193, for the times indicated. Nuclear protein extracts were prepared and 5 μg was subjected to EMSA analysis using a ^32^P labelled HIV-1 κB oligonucleotide.

We first investigated the ability of different topoisomerase II inhibitors to induce NF-κB DNA-binding. Previously we have shown that daunorubicin and UV-C induce NF-κB DNA-binding with delayed kinetics relative to an inducer such as TNF [7]. Together with other data in the literature suggesting differences in their mechanism of IKK activation, this has lead us to designate daunorubicin and UV-C as atypical inducers of NF-κB while TNF is a prototypical example of a typical inducer [7,24]. In addition, we have also shown previously that etoposide is also a strong inducer of NF-κB DNA-binding in U-2 OS cells [8]. Consistent with this previous data, doxorubicin, which is structurally very similar to daunorubicin [12], also induced NF-κB DNA-binding with delayed kinetics (Fig. 1). Interestingly, mitoxantrone, which does not undergo redox cycling and therefore does not produce free radicals [16], was also a strong inducer of NF-κB DNA-binding (Fig. 1). Indeed, induction of NF-κB DNA-binding occurred more rapidly than with doxorubicin, suggesting that if anything, under these conditions, oxygen free radicals delay NF-κB activation, in contrast to their previously ascribed role as cell type specific inducers of NF-κB [18-20]. Significantly, aclarubicin, which intercalates with DNA [9], also induced NF-κB DNA-binding while ICRF-193, which inhibits topoisomerase II but does not directly induce DNA-damage [22,23], did not (Fig. 1). These observations are summarized in Table 1.

### Production of oxygen free radicals is not required for induction of NF-κB DNA-binding by anthracyclines

To confirm the apparent lack of a requirement for oxygen free radical generation for induction of NF-κB DNA binding, implied by the effect of mitoxantrone, we investigated the effect of reducing agents. Although reactive oxygen species have been shown to activate NF-κB in a cell context dependent manner [19,20], it has been recently shown that many of the compounds used in studies to neutralize free radicals in themselves could inhibit NF-κB activity. For example, N-acetyl-L-cysteine (NAC) and pyrrolidine dithiocarbamate (PDTC), anti-oxidants commonly used in studies of NF-κB activation, prevented TNF induced NF-κB DNA binding independent of their antioxidant activity [25]. NAC was shown to lower the affinity of TNF for its receptor, whilst PDTC inhibited the IκB-ubiquitin ligase activity. In contrast, alternative antioxidants such as epigallocatechingallate (EGCG), the main component of green tea, and trolox, a soluble vitamin E analogue (both strong phenolic radical scavengers) did not affect TNF induced NF-κB activation [25].

**Figure 2:**
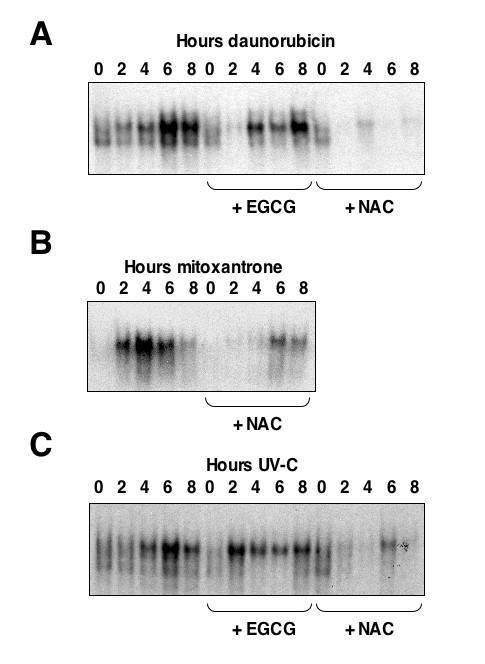
Oxygen free radical generation is not required for induction of NF-κB DNA-binding. U-2 OS cells were stimulated with (A) 1 μM daunorubicin, (B) 2.5 μM mitoxantrone (C) 40 J/m^2 ^UV-C, or for the indicated times with, or without 1 hour pre-incubation with 20 μM EGCG or 10 mM NAC as indicated.

We therefore compared the ability of both EGCG and NAC to inhibit daunorubicin induced NF-κB DNA-binding. Consistent with our observation that mitoxantrone is a strong inducer, EGCG had no significant effect on NF-κB DNA-binding (Fig. 2A). In contrast, NAC inhibited both daunorubicin and, interestingly, mitoxantrone induced NF-κB DNA-binding (Fig. 2A & B). This latter effect confirms that NAC is a free-radical independent inhibitor of NF-κB. We also investigated the effect of these compounds on UV-C induced NF-κB DNA-binding, since free-radical generation is also a component of the effect of this stimulus. Similarly, EGCG did not inhibit UV-C induced NF-κB DNA-binding while NAC did (Fig. 2C). In this experiment EGCG treatment appeared to result in earlier induction of NF-κB by UV-C, more akin to that seen with mitoxantrone, suggesting that oxygen free radicals might affect the kinetics of NF-κB activation. Consistent with this, etoposide, which also does not induce free radicals, similarly induces NF-κB more rapidly [8].

These results, together with our previous data, suggest that DNA-damage is required for induction of NF-κB DNA-binding and that this can occur either through DNA-intercalation or topoisomerase II inhibition. But neither topoisomerase II inhibition per se, or production of oxygen free radicals are required for this effect. Of course, we cannot rule out some other, unknown, DNA-damage independent, effect of these drugs that also influences these processes.

### Topoisomerase II inhibition does not induce NF-κB dependent transcriptional repression

**Figure 3:**
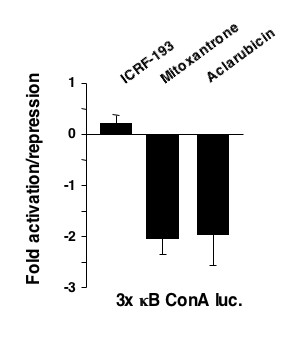
Mitoxantrone and aclarubicin but not ICRF-193, repress NF-κB reporter plasmid activity. U-2 OS cells were transfected with 2 μg of 3 × κB ConA luciferase reporter plasmid and 36 hours later stimulated with 4 μM ICRF-193, 1 μM aclarubicin or 2.5 μM mitoxantrone for 8 hours. Results are normalised such that no change in luciferase activity has a value of 0.

We next investigated effects on transcriptional activity. Significantly, and similar to our previously observed effects with daunorubicin and UV-C [7], both mitoxantrone and aclarubicin treatment repressed the activity of a 3 × κB reporter plasmid despite being inducers of NF-κB DNA-binding, (Fig. 3). We had previously found that cisplatin treatment of U-2 OS cells, while not inducing NF-κB DNA-binding does modulate the activity of the basal level of active NF-κB found in these cells [8]. Therefore, although ICRF-193 did not induce DNA-binding by NF-κB, we also determined whether it would have any effect on transcription. In this case however, ICRF-193 did not affect the activity of either the 3 × κB reporter plasmid or Bcl-xL and XIAP (Fig. 3 and see also 4C). Therefore, inhibition of topoisomerase II alone does not appear to affect NF-κB function in U-2 OS cells.

### Oxygen free radicals do not induce NF-κB dependent transcriptional repression

**Figure 4:**
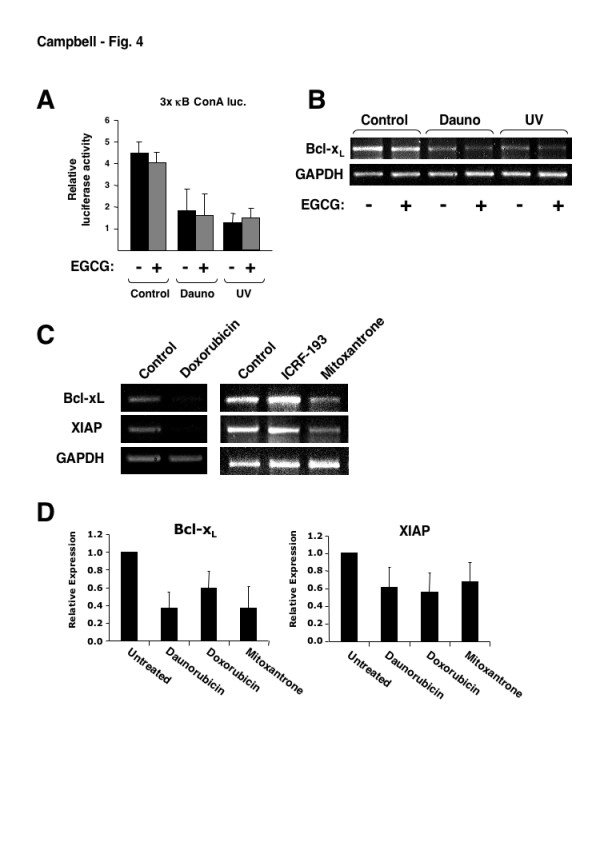
Oxygen free radical generation is not required for NF-κB dependent repression of transcription. (A) U-2 OS cells were transfected with 2 μg of 3 × κB ConA luc and 36 hours after transfection cells were unstimulated (control) or stimulated for 8 hours with 1 μM daunorubicin or 40 J/m^2 ^UV-C, with, or without, 1 hour pre-stimulation with 20 μM EGCG, as indicated. (B) U-2 OS cells were unstimulated (control) or stimulated for 6 hours with 1 μM daunorubicin or 40 J/m^2 ^UV-C, with, or without, 1 hour pre-stimulation with 20 μM EGCG, as indicated. Total RNA was prepared and semi-quantitative PCR analysis with primers specific to human Bcl-xL and GAPDH. (C) Mitoxantrone inhibits Bcl-xL and XIAP mRNA levels. U-2 OS cells were stimulated for 6 hours with 4 μM ICRF-193, 2.5 μM mitoxantrone and 1 μM doxorubicin. Total RNA was prepared and semi-quantitative PCR analysis performed using primers to human Bcl-xL, XIAP and GAPDH control. (D) Quantitative RT-PCR analysis. The effects of danuorubicin, doxorubicin and mitoxantrone on Bcl-xL and XIAP mRNA levels was confirmed by quantitative real-time PCR analysis.

Although oxygen free radicals are not required for induction of NF-κB DNA-binding by topoisomerase II inhibitors, it remained possible that they might affect transcriptional activity. We therefore investigated the effect of EGCG on daunorubicin induced NF-κB dependent repression of transcription. No effect was seen, however, with both the 3 × κB reporter plasmid or upon endogenous Bcl-xL expression (Fig. 4A & B). Identical results were also seen with UV-C (Fig. 4A & B). In addition, consistent with the result seen in Fig. 3, mitoxantrone still repressed the expression of endogenous Bcl-xL and XIAP (Fig. 4C). Repression of endogenous Bcl-xL and XIAP expression by daunorubicin, doxorubicin and mitoxantrone was confirmed by quantitative RT-PCR analysis (Fig. 4D). Therefore, taken together, oxygen free radicals do not appear to affect NF-κB transcriptional activity induced by topoisomerase II inhibitors and UV-C in U-2 OS cells.

## Discussion

The results in this report, together with our previous published observations [7,8] highlight the diversity of the NF-κB response to different chemotherapeutic drugs and cytotoxic stimuli in U-2 OS cells. Some stimuli, such as TNF and etoposide, induce both NF-κB DNA-binding and target gene expression. Other stimuli, however, such as the topoisomerase II inhibitors daunorubicin, doxorubicin, mitoxantrone and aclarubicin, together with ultraviolet light, induce NF-κB DNA-binding but result in the repression of NF-κB dependent gene expression. Other pathways, induced by cisplatin and the ARF tumor suppressor, do not appear to affect NF-κB nuclear localization but rather modulate the activity of NF-κB, resulting in a more selective repression of NF-κB target genes [8]. These effects imply multiple NF-κB regulatory pathways and that NF-κB subunit transactivation can be a separate regulatory event to nuclear translocation. Here, we have focused on the different known characteristics of anthracyclines and other topoisomerase II inhibitors to begin to determine which of these features are critical for specific effects on NF-κB function.

It is apparent that DNA-damage per se does not induce a defined effect on NF-κB function. As these compounds can induce DNA-lesions in different ways and also have other cellular effects, we were interested in which of these were required for NF-κB regulation. A straightforward interpretation of our data would indicate that those compounds capable of intercalating with DNA all induce NF-κB DNA-binding together with repression of NF-κB reporter plasmids and the target genes Bcl-xL and XIAP (Table 1). By contrast, when topoisomerase II inhibition results in DNA damage, as is seen with etoposide, induction NF-κB DNA-binding is seen but with activation rather than repression of NF-κB transactivation [8]. Although some compounds are capable of both intercalating with DNA and inhibiting topoisomerase II (Table 1) we have previously observed that transcriptional repressive effects on NF-κB are dominant over transcriptional activatory effects [7,8], possibly accounting for why, with these compounds we see repression. Furthermore, DNA-damage appears to be a requirement for effects on NF-κB since ICRF-193 treatment results in topoisomerase II inhibition alone and here we see no effect (Table 1).

This might be an overly simplistic interpretation, however, since Bilyeu and co-workers recently showed that DNA damage was not necessary for anthracycline induced NF-κB activation [26]. To do this they utilized cytoplasmically localized anthracycline derivatives, which surprisingly still induce NF-κB DNA-binding. Immobilized (extracellular) or free (intracellular) anthracyclines, have also been reported to activate NF-κB DNA binding, yet immobilized daunorubicin and doxorubicin induced necrosis rather than apoptosis, the effect typically seen with these compounds [27]. These studies raise the question of whether DNA damage is in fact required for induction of NF-κB activity by topoisomerase II inhibitors. Furthermore, some reports do suggest that ICRF-193 treatment will result in DNA-damage as a secondary consequence of topoisomerase II inhibition [28], underlining that the effects of these compounds are often somewhat overlapping and caution should be taken interpreting there effects.

Another feature of some anthracyclines is their ability to generate oxygen free radicals resulting from redox cycling. We demonstrated, however, through the use of mitoxantrone and the free radical neutralizer EGCG that this effect is not required for induction of NF-κB DNA-binding and repression of NF-κB target genes (Table 1). DNA-binding assays with both mitoxantrone and EGCG treated cells did suggest that oxygen free radicals might affect the timing of NF-κB induction, with free radical production potentially inducing a delay in the kinetics of NF-κB activation. The mechanism for this is unknown and will require further research but could involve direct effects on the dimerisation and DNA-binding of NF-κB subunits themselves, which have been shown to possess reactive cysteine residues sensitive to cellular redox status [29]. Interestingly, NAC, which has been shown to inhibit NF-κB through pathways independent of its ability to function as a reducing agent [25], also inhibited NF-κB activity induced by daunorubicin and UV-C while EGCG did not. This suggests that the non-specific inhibitory effects of this compound are quite broad and previous results obtained with it should be treated with caution.

One possibility is that all the effects of these compounds, DNA intercalation, topoisomerase II inhibition and free radical generation, affect NF-κB and are capable of inducing its nuclear translocation and DNA-binding separately. This might also account for the effects seen by Bilyeu et al., which could derive from cytoplasmic effects or oxygen free radical production. However, it is likely that the functional consequences of these different characteristics are different. In this regard, it is DNA-intercalation and the subsequent effects it induces that result in repression of NF-κB dependent transcription. This implies that DNA-damage induced by this pathway has effects distinct to those seen by other forms of DNA-damage. The precise nature of these will be the subject of future investigations. Our previous results also suggest that the pathways that result in NF-κB dependent repression of transcription induced by UV-C and cisplatin/ARF are different from each other and also those induced by topoisomerase II inhibitors and DNA-intercalators. Therefore, there is likely to be much complexity in the pathways that regulate the function of NF-κB subunits after they translocate to the cell nucleus. However, since these pathways are being induced by drugs commonly used in cancer therapy, understanding them could be of great diagnostic and prognostic value. As NF-κB can exhibit such a diverse set of responses to such treatments, care should be taken when predicting its role in tumorigenesis and the response to chemotherapeutic drug therapy.

## Conclusion

Induction of NF-κB DNA-binding and transcriptional repression by clinically utilized topoisomerase II inhibitors and DNA-intercalators correlates with their ability to intercalate into DNA and not with topoisomerase II inhibition or oxygen free radical production. These latter effects have been shown to affect NF-κB function in other circumstances but our data implies that the particular signaling events initiated by the DNA-damage resulting from helical torsion generated by DNA-intercalation, specifically target the transactivation functions of NF-κB. Our previous data indicates that this will involve the differential phosphorylation of the RelA(p65) NF-κB subunit [24].

These different characteristics and effects on NF-κB could have important clinical effects and also impact future drug design. Drugs that result in forms of NF-κB that repress anti-apoptotic gene expression might be more clinically effective. The challenge will be to be able to predict which effects will be seen in patients since these effects are likely to be both cell and tumour type specific. Nonetheless, there is the potential for the development of new diagnostic and prognostic tools. It is interesting to note that doxorubicin and cisplatin, both of which, in U-2 OS osteosarcoma cells, result in NF-κB dependent repression of anti-apoptotic target genes through different mechanisms [7,8] are used in combination for the treatment of osteosarcoma in the clinic [13]. Whether these effects contribute to their effectiveness in the clinic and whether patients who do not respond to therapy have lost these NF-κB regulatory pathways, will require further research. It is also possible, that further knowledge of these pathways can help in future drug design and discovery.

## Methods

### Cells

U-2 OS osteosarcoma cells were obtained from the European Collection of Cell Cultures and were not grown beyond passage 35. Cell were grown at 37°C with 5% CO_2 _in Dulbecco's Modified Eagles Medium (DMEM) containing 10% Fetal Bovine serum and antibiotics. Where indicated, endogenous NF-κB activity was induced by 1 μM aclarubicin (Sigma), 1 μM daunorubicin (Affiniti), 1 μM doxorubicin (Affiniti), 2.5 μM mitoxantrone (Sigma) and 4 μM ICRF-193 (Affiniti) or 40 J/m^2 ^UV-C (254 nm) using a Stratalinker (Stratagene).

### Quantitative Real Time (RT) PCR

RNA was extracted from untreated cells or cells treated with daunorubicin, doxorubicin or mitoxantrone using the Nucleospin II kit (Machery-Nagel) according to manufacturer's instructions. 10 ng RNA was used per reaction. Quantative RT-PCR was performed using the SuperScript™ III Platinum^® ^SYBR^® ^Green One-Step qRT-PCR Kit (Invitrogen) together with gene specific QuantiTect^® ^Primers (Qiagen), as per manufacturers instructions. Data was generated on a Rotor-Gene 3000 (Corbett Research) using the following experimental settings: Hold 50°C for 3 min; Hold 95°C 5 min; Cycling (95°C for 15 sec; 55°C for 30 sec; 72°C for 15 sec with fluorescence measurement) × 45; Melting Curve 50–99°C with a heating rate of 1°C every 5 sec. All values were calculated relative to untreated levels and normalised to GAPDH levels using the Pfaffl method [30]. Each RNA sample was assayed in triplicate and the results shown are representative of three separate experiments.

### Other experimental procedures

All reporter plasmid luciferase assays, semi-quantitative PCR and electrophoretic mobility shift assays (EMSA) were performed as described previously [7,31,32]. All plasmids and PCR primers siRNAs have been described and characterized before [7,31,32]. Luciferase assays were performed according to the manufacturer's instructions (Promega) and results were normalized for protein concentration with all experiments performed a minimum of three times before calculating means and standard deviation as shown in the figures.

## Authors' contributions

KJC performed and designed all experiments and contributed to manuscript writing.

JMOS helped with further experimentation and revisions to the manuscript.

NDP contributed to experimental design and manuscript writing.

All authors read and approved the final version of the manuscript.

## Pre-publication history

The pre-publication history for this paper can be accessed here:



## Figures and Tables

**Table 1:** Summary of the different characteristics of the compounds used in this study and their effect on NF-κB. Our previous work on the effects of daunorubicin and etoposide on NF-κB are described in references [7, 8]. * Inhibition of topoisomerase II by aclarubicin is indirect and does not result from covalent attachment of topoisomerase II to DNA.

	Topo II inhibition	Topo II inhibition induced DNA-damage	DNA intercalation	Generation of oxygen free radicals	Induction of NF-κB DNA-binding	Repression or activation of transcription
Daunorubicin	Yes	Yes	Yes	Yes	Yes	Repression
Doxorubicin	Yes	Yes	Yes	Yes	Yes	Repression
Mitoxantrone	Yes	Yes	Yes	No	Yes	Repression
Aclarubicin	Yes*	No	Yes	Yes	Yes	Repression
ICRF 193	Yes	No	No	No	No	No effect
Etoposide	Yes	Yes	No	No	Yes	Activation
